# Supplementary material for: A biophysical model of viral escape from polyclonal antibodies
Source: Virus Evol. 2022 Dec 12;8(2):veac110. doi: 10.1093/ve/veac110 (PMC9793855; doi:10.1093/ve/veac110)
Supplement: veac110_Supp [file veac110_supp.zip › Appendix.docx]

# Appendix

We have described a biophysical model that assumes a polyclonal antibody mixture can be divided into independent groups of antibodies that bind to distinct epitopes without competition. Here we interrogate the validity of this assumption, being cognizant of the observation that realistic viral epitopes are often overlapping and therefore not distinct. To do this, we draw from statistical mechanics principles to compare the antibody escape fractions predicted by our independent epitope model and an identically formulated model that instead assumes all epitopes are overlapping.

***1. Monoclonal antibody case***

Before considering the polyclonal antibody case, we first consider the case of a monoclonal antibody that binds a viral antigen. Here, the viral protein can exist in two microstates: bound or unbound by the antibody. The Boltzmann weight is 1 for the unbound state and $\frac{c}{K_{d}}$ for the bound state, where $c$ is the antibody concentration and $K_{d}$ is the dissociation constant of antibody-antigen binding. These weights can be derived using the steady-state approximation (Einav and Bloom 2020). We can then define the partition function $\Xi$ as:

$$\Xi=\sum_{i} Z_{i}=Z_{\text{unbound}}+Z_{\text{bound}}$$

where $Z_{i}$ represents the Boltzmann weights of the $i$ microstates. Given this, the probability of a viral antigen being unbound is:

$$p_{\text{unbound}}=\frac{Z_{\text{unbound}}}{\Xi}=\frac{1}{1+\frac{c}{K_{d}}}$$

***2. Polyclonal antibody case***

For a polyclonal antibody mixture, we modify $c$ to represent the concentration of the polyclonal antibody mixture. We assume that the polyclonal antibody mixture contains antibodies that bind one of $E$ epitopes. As follows, the Boltzmann weight of the state where epitope $e$ is bound is modified to $\frac{cf_{e}}{K_{d,e}}$, where $f_{e}$ represents the fraction of antibodies in the mixture that target epitope $e$, and $K_{d,e}$ is the dissociation constant of antibodies binding to epitope $e$.

***2.1 Two distinct epitopes***

In a polyclonal antibody mixture, new microstates exist where multiple epitopes are bound by antibodies. For example, we can consider a viral antigen that contains two distinct epitopes (1 and 2) that are targeted by polyclonal antibodies. In addition to the microstates where a single epitope is bound, we now require an additional microstate where both epitopes are bound. The Boltzmann weight for this new microstate is:

$$Z_{12,\text{bound}}=\left( \frac{cf_{1}}{K_{d,1}} \right)\left( \frac{cf_{2}}{K_{d,2}} \right)$$

We can then rewrite the partition function $\Xi$ as:

$$\Xi=\sum_{i} Z_{i}=Z_{\text{unbound}}+Z_{1,\text{bound}}+Z_{2,\text{bound}}+Z_{12,\text{bound}}$$

and the probability of a viral antigen being unbound is:

$$p_{\text{unbound}}=\frac{Z_{\text{unbound}}}{\Xi}=\frac{1}{1 + \frac{cf_{1}}{K_{d,1}} +\frac{cf_{2}}{K_{d,2}}+\left( \frac{cf_{1}}{K_{d,1}} \right)\left( \frac{cf_{2}}{K_{d,2}} \right)}$$

$$=\left( \frac{1}{1 + \frac{cf_{1}}{K_{d,1}}} \right) \left( \frac{1}{1 + \frac{cf_{2}}{K_{d,2}}} \right)$$

Note that this is the biophysical model that is described in the main text.

***2.2 Two overlapping epitopes***

In 2.1, the two epitopes were distinct and there was no competition amongst antibodies. However, if the epitopes are overlapping and there is competition, then the microstate where both epitopes are bound ($Z_{12, \text{bound}}$) can no longer exist. In this case, the probability of a viral antigen being unbound is:

$$p_{\text{unbound}}=\frac{Z_{\text{unbound}}}{\Xi}=\frac{1}{1 + \frac{cf_{1}}{K_{d,1}} +\frac{cf_{2}}{K_{d,2}}}$$

***2.3 Extending beyond two epitopes***

The same logic applies to viral antigens with more than two epitopes targeted by antibodies. For example, we can write $p_{\text{unbound}}$ for the case of three distinct epitopes as:

$$p_{\text{unbound}}=\frac{Z_{\text{unbound}}}{\Xi}=\frac{1}{1 + \frac{cf_{1}}{K_{d,1}} +\frac{cf_{2}}{K_{d,2}}+\frac{cf_{3}}{K_{d,3}}+\left( \frac{cf_{1}}{K_{d,1}} \right)\left( \frac{cf_{2}}{K_{d,2}} \right)+\left( \frac{cf_{1}}{K_{d,1}} \right)\left( \frac{cf_{3}}{K_{d,3}} \right)+\left( \frac{cf_{2}}{K_{d,2}} \right)\left( \frac{cf_{3}}{K_{d,3}} \right)+\left( \frac{cf_{1}}{K_{d,1}} \right)\left( \frac{cf_{2}}{K_{d,2}} \right)\left( \frac{cf_{3}}{K_{d,3}} \right)}$$

$$=\left( \frac{1}{1 + \frac{cf_{1}}{K_{d,1}}} \right) \left( \frac{1}{1 + \frac{cf_{2}}{K_{d,2}}} \right)\left( \frac{1}{1 + \frac{cf_{3}}{K_{d,3}}} \right)$$

and the case of three overlapping epitopes as:

$$p_{\text{unbound}}=\frac{Z_{\text{unbound}}}{\Xi}=\frac{1}{1 + \frac{cf_{1}}{K_{d,1}} +\frac{cf_{2}}{K_{d,2}}+\frac{cf_{3}}{K_{d,3}}}$$

Overall, we found that the predicted $p_{\text{unbound}}$ does not differ by much between the independent and overlapping epitope models under realistic $c$’s, $f$’s, and $K_{d}$’s. However, the predicted $p_{\text{unbound}}$ becomes more discordant between the two models as the number of epitopes increases, given the number of microstates with multiply bound epitopes increases. In practice, we only fit models to a handful of epitopes and not all of them will overlap. As such, we maintain that the independent epitope assumption is appropriate. To interactively explore how $p_{unbound}$ differs between the two models, see <https://jbloomlab.github.io/polyclonal/partition_function.html>
