# Supplementary figures and images for: A biophysical model of viral escape from polyclonal antibodies
Source: Virus Evol. 2022 Dec 12;8(2):veac110. doi: 10.1093/ve/veac110 (PMC9793855; doi:10.1093/ve/veac110)

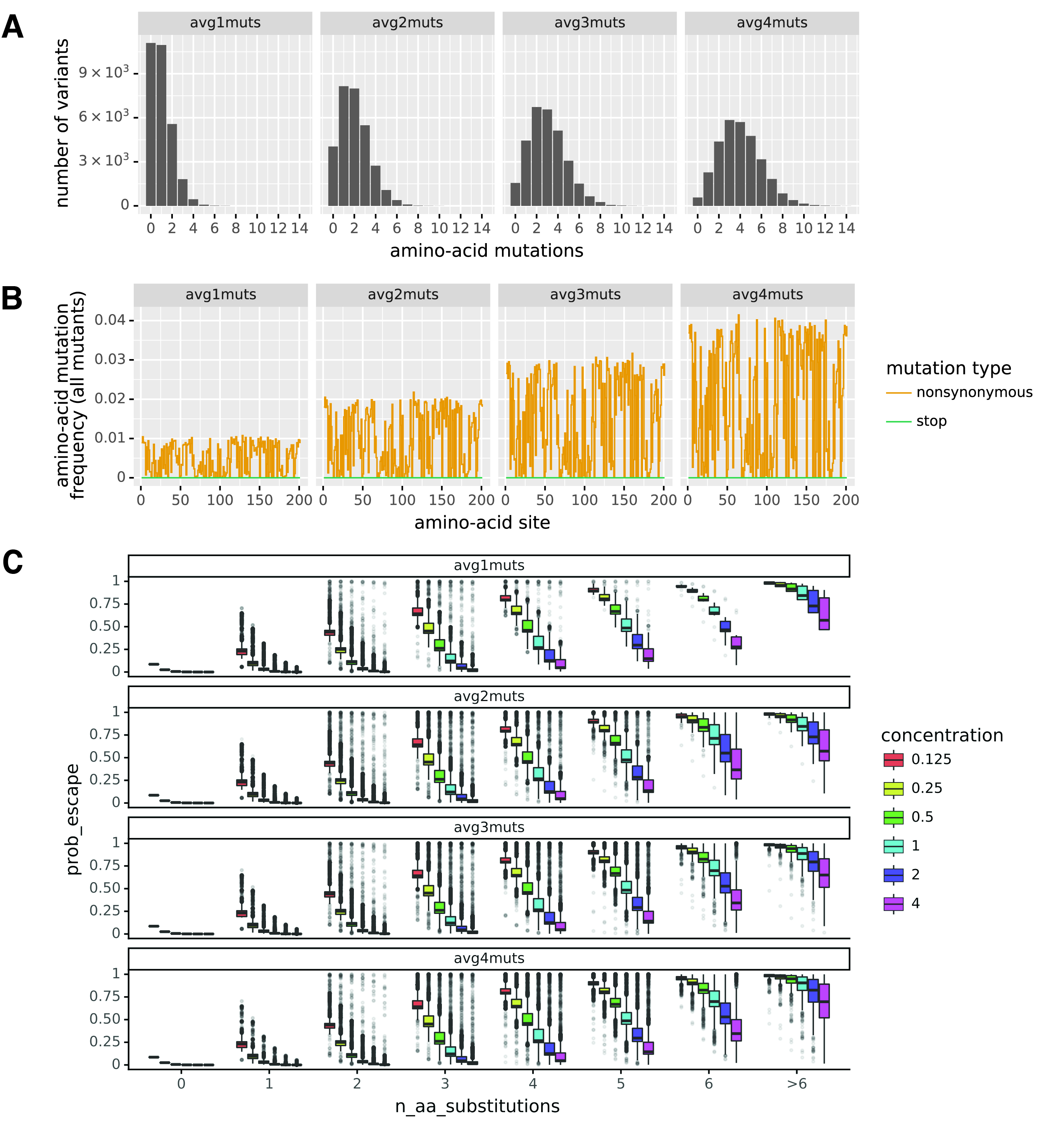

Supplement: veac110_Supp [file veac110_supp.zip › figureS1.tif]
